# Cognitive and Emotional Dysfunction after Central Pontine Myelinolysis

**DOI:** 10.1155/2003/872916

**Published:** 2003-01-01

**Authors:** Tatia M. C. Lee, Crystal C. Y. Cheung, Esther Y. Y. Lau, Amanda Mak, Leonard S. W. Li

**Affiliations:** ^1^Neuropsychology LaboratoryThe University of Hong KongHong Kong; ^2^Institute of Clinical NeuropsychologyThe University of Hong Kong and MacLehose Medical Rehabilitation CentreHong Kong; ^3^Department of MedicineThe University of Hong KongHong Kong

**Keywords:** Central Pontine Myelinolysis, pathological laughing and crying, cognition, emotion

## Abstract

The case of a 67-year-old right-handed Chinese man with Central Pontine Myelinolysis [CPM] is described to illustrate the resulting cognitive and emotional disturbances. A comparison of the data in this report with that in published studies suggests that ethnicity does not seem to have much effect on the symptoms of CPM. Possible underlying neural-pathological mechanisms are discussed. This case further substantiates the speculation that the brainstem plays a role in higher cognitive processes and emotional regulation.

## 1. Cognitive and emotional dysfunction after central pontine myelinolysis

Central pontine myelinolysis (CPM), though rare, has increasingly been recognized as a neurological disorder associated with a lesion of the central portion of the basis pontis [1]. Possible sites of lesion include the corticospinal, corticobular, and pontocerebellar tracts; cranial nerves; and other ventral pontine structures [2]. Adam et al. suggested that such lesions could not be accounted for by ischaemia, hypoxia, or temporal lobe herniation [3]. However, some researchers suggest a causal role of hypoxia in CPM, while others speculate that CPM is the result of the rapid correction of electrolyte disturbances, particularly hyponatraemia [4].

The role of the brainstem in human cognition and emotion remains a mystery. Animal studies suggest that the brainstem plays a role in learning, memory, and attention [5,6]. It has been difficult to systematically study the relationship between brainstem lesions and human cognition and emotion, partly because of the high mortality associated with circumscribed brainstem infarcts, and partly because of the rarity of conditions like CPM. Here, we describe the case of a surviving CPM victim to provide some insight into the possible role of the brainstem in higher cognitive processes and emotional regulation. Ethnical differences in symptoms after CPM between Chinese and Caucasians are explored.

## 2. Case background

The patient was a 67-year-old right-handed Chinese businessman with undergraduate education in Mainland China. His medical history was uneventful until this admission for emergency liver transplantation owing to acute liver failure. Post-operatively, the patient developed intra-abdominal bleeding, respiratory failure, renal failure, and liver abscess. His acute medical condition gradually settled down, but he developed hypernatraemia and was unarousable for three months. A brain MRI (Fig. 1) with T2-weighted image revealed

ISSN 0953-4180/03/$8.00 © 2003 – IOS Press. All rights reserved hyperintense signal in pons. No other lesion was detected at medulla, cortical or subcortical structures on the MRI except mild cerebral atrophy which was compatible with patient's age. The patient was diagnosed with CPM with no extra-pontine involvement on MRI findings. His post-operative complications gradually settled down and he regained consciousness. He had residual weakness on all four limbs for which he was transferred for intensive rehabilitation 6 months after the acute event. He had no cerebellar signs clinically but had mild dysarthria which was most likely due to mild weakness of facial muscles and muscles of mastication. The patient subsequently had good motor recovery and was independent in walking and activities of daily activities. His renal and liver function tests [Sodium 141(136–148), Potassium 4.1(3.6–5.0), Urea 6.2(3.1–7.4), Creatinine 108(55–105), Bilirubin 5(3–22), Albumin 35g/L(35–51), ALP 133U/L(38–126), AST 21U/L(15–46) and ALT 12U/L(11–66)] had already returned to normal during the period of rehabilitation. About six months after operation, six 1-hour neuropsychological assessment sessions using culturally fair and validated tests were conducted. At that moment, his medications were Lamivadine, Prednisolone, Pepcidine and Tarcolimus, all of which should not affect his cognitive function. The patient was cooperative and effortful in testing. His performance was compared with Chinese normative data [7–9] (Table 1). A validated Pathological Laughing and Crying Scale was also administered [10] since emotional lability, which seems to be a common manifestation of brain damage [11,12], was observed in this patient.

On presentation, the patient was alert and oriented to time, place, and person. His speech was relevant, and coherent, though slightly slurred. He was able to comprehend test instructions and follow conversations. No mood disturbances and personality changes were reported by the patient or the family. Nonetheless, episodes of emotional lability were observed by the family and during testing.

The patient's performance on the administered neuropsychological tests was mostly impaired. His intellectual ability as estimated by the Test of Nonverbal Intelligence, 3rd edition, was below average and was much lower than what would be expected from his premorbid academic and vocational achievements. Though his visual attention and visual-spatial judgment were within the average range, deficits in all other cognitive abilities assessed were observed. His auditory attention span was impaired. His divided attention was weak, aggravated with increased cognitive load. As for selective attention, his extreme slowness greatly affected his performance, though he did demonstrate an ability to inhibit strongly competing yet irrelevant distractions. The patient could acquire and retain new information, but with a flatter learning curve than expected. Verbal and nonverbal frontal fluency was impaired. His tendency to perseverate and his impaired ability in using feedback to modify his cognitive strategies rendered him less capable in problem solving.

On the Pathological Laughing and Crying Scale, the patient's responses indicated uncontrollable crying spells more often than laughing episodes. The patient reported crying without reason and excessive sadness emotional expressions, leading to his fear of losing emotional control.

## 3. Discussion

### *3.1. Cognition*

Except for visual attention span and visuo-spatial judgment, this patient suffered from deficits in all other cognitive functions tested, suggesting that abilities in which the posterior right hemisphere plays a major role might be better preserved.

The role of pontine pathology in cognitive function remains unclear. Disruption of the corticopontine networks is speculated as one possible cause. Indeed, animal studies demonstrate that preliminary processing of information takes place in the brainstem prior to its transfer to the cortex [13]. Furthermore, cortical endings of pontine projections appear most prominent in the dorsolateral prefrontal area [14], which is crucial not only for motor planning but also other "frontal" cognitive functions including memory. Therefore, pontine lesions may disrupt the neural pathways emanating from the brainstem resulting in dysfunction upstream [15].

Another hypothesis involves the interruption of neurotransmitter pathways – most likely dopaminergic and cholinergic pathways – that emerge from the brainstem, and have an impact on cognitive operations [16]. Other reports show that reduced choline acetyltransferase in the mesopontine tegmentum correlates with decreased cognitive functioning in people with schizophrenia [17].

A review of the few case reports of CPM and the present case indicates that impaired global cognitive abilities and emotional lability are universal features of CPM. The Chinese patient in this case, however,

Table 1
Neuropsychological Assessment: Tests Administered and the Results

| | Test | Z-score |
|---|---|---|
| Intelligence | Test of Nonverbal Intelligence – 3rd edition | $< -2.13$ |
| Attention | Digit Span Test – Forward | $-2.52$ |
| | Visual Span Test | 0.25 |
| | The Balloon Test | NAD |
| | Color Trails Test – Part I | $-0.41$ |
| | Part II | $-2.28$ |
| | Stroop Color Word Test – Dots | $< -3.00$ |
| | Words | $< -3.00$ |
| | Colors | $< -3.00$ |
| | Symbol Digits Modality Test – Written | $-1.31$ |
| | Oral | $-1.43$ |
| Memory | Chinese Auditory Verbal Learning Test | |
| | – Trial 1 | $-1.16$ |
| | – Trial 2 | $-1.98$ |
| | – Trial 3 | $-1.70$ |
| | – Trial 4 | $-2.50$ |
| | – Trial 5 | $-1.23$ |
| | – Interference Trial | $-2.20$ |
| | – Immediate Recall | $-0.44$ |
| | – 30-min Delayed Recall | $-0.47$ |
| | – Recognition | $-1.20$ |
| | Recognition Memory Test for Faces | $< -3.00$ |
| Visuo-spatial | Judgment of Line Orientation Test Hooper Visual Organization Test | $-0.27$ |
| Functions | Hooper Visual Organization Test | $< -3.00$ |
| Frontal Functions | Verbal Fluency Test | |
| | – Fruits and Vegetables | $-1.36$ |
| | – Animals | $-2.23$ |
| | Ruff Figural Fluency Test | |
| | – Unique Design | $-2.89$ |
| | – Error Ratio | $-1.76$ |
| | Wisconsin Card Sorting Test | |
| | – Perseverative responses | $< -3.00$ |
| | – Conceptual level responses | $< -3.00$ |
| | – Number of categories completed | $< -3.00$ |

seems to have better memory, visual attention span, and visuo-spatial judgment ability. This variance in symptomatic presentation is probably the result of the different co-morbid medical conditions like hypoxia and periventricular white matter disease [1].

### 3.2. *Emotion*

The phenomenon of pathological laughter and crying is a well-known correlate of many neuropathologic syndromes [18]. Consistent with previous reports of CPM [19], emotional lability, especially crying, was observed in this case. It was once thought that the emotional lability observed was secondary to corticobulbar lesions [20]. However, Asfora et al. [21] reported that disinhibition of the emotional apparatus could be due to a lesion that did not clinically derange corticobulbar tracts. This observation is in line with Wilson's [22] speculation that there may exist an upper pontine supranuclear facio-respiratory center integrating both facial and respiratory mechanisms for emotional expression and controlling corticofugal paths.

On the other hand, Mendez et al. [23] reported a case of disorder of laughter after the rupture of an anterior communicating artery aneurysm with subsequent bifrontal injury from surgical intervention. Our patient presented with more pathological crying than laughter. It is highly possible that pathological crying and laughter could be two dissociable phenomena subserved by distinct neuroanatomical circuitary. The development of a comprehensive theoretical model of pathological laughter and crying is important for an integrative understanding of its mechanism.

Other possible underlying mechanisms of emotional lability seen in CPM could be the disruption of the ascending projections of the raphe nuclei, pontomesencephalic reticular formation, and perhaps even those of the nucleus locus coeruleus. This would interfere with the cortical and thalamic supply of serotonin, acetylcholine, and norepinephrine, leading to disruption of

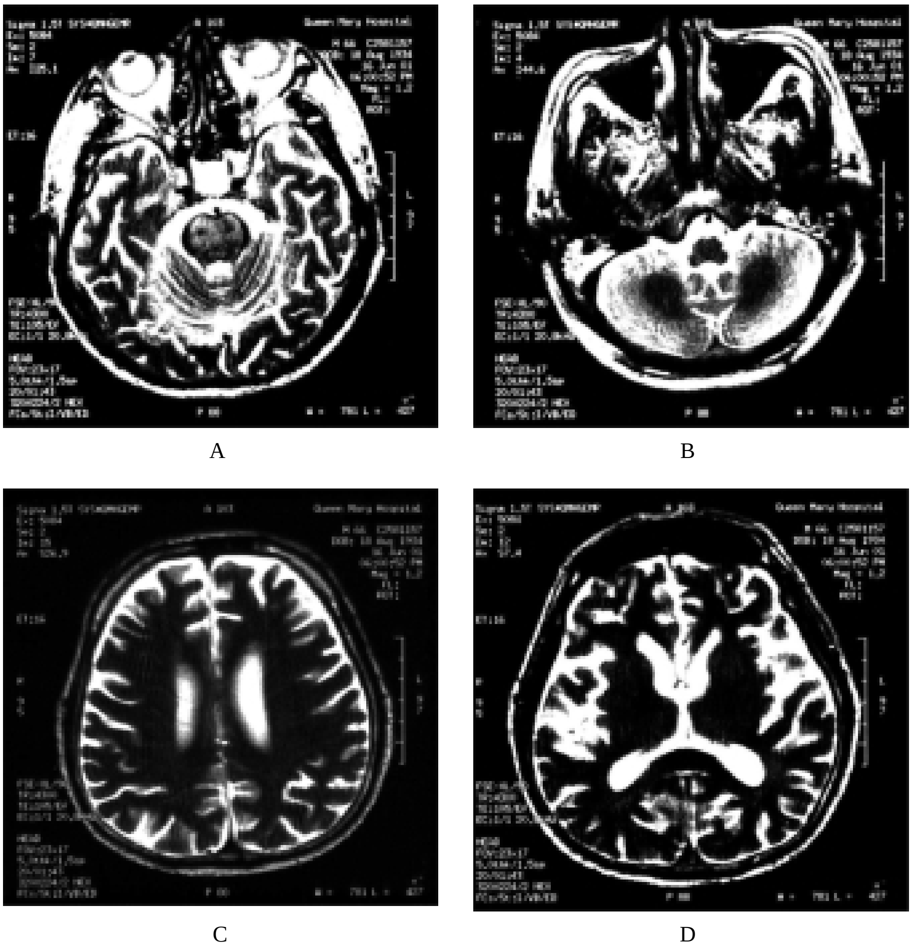

Fig. 1. Axial T2-weighted MR Scan of the patient showed symmetrical area of abnormal high signal intensity in the basis pontis (A) but not in the medulla (B), cortical (C) and subcortical (D) regions.

mood and arousal modulation [24]. Indeed, there have been reports of successful treatment of pathological crying using the selective serotonin reuptake inhibitor citalopram [25]. Antidepressants was also shown to be effective in treating emotional lability [10,26], suggesting that the disorder may involve dysfunction of the neural biogenic amine systems. Bridgeford et al. [27] indicate the effectiveness of methylphenidate in reversing the neuropsychiatric symptoms associated with central and extrapontine myelinolysis.

## 4. Conclusion

This case study demonstrates the possible role of the brainstem in higher cognitive processes and emotional regulation. Disruption of the corticopontine networks as well as the neurotransmitter pathways, together with the complication of other medical conditions with CPM, may cause the pattern of deficits in the form of lowered global cognition and impaired higher cortical functions. In addition, pathological emotional spells – crying more than laughter – were prominent behavioral manifestations [28]. A comparison of this report with published studies suggests that symptomatic presentation of CPM is universal with minimal ethnical differences.
